# FGF23, a novel muscle biomarker detected in the early stages of ALS

**DOI:** 10.1038/s41598-021-91496-6

**Published:** 2021-06-08

**Authors:** Ying Si, Mohamed Kazamel, Michael Benatar, Joanne Wuu, Yuri Kwon, Thaddaeus Kwan, Nan Jiang, Dominik Kentrup, Christian Faul, Lyndsy Alesce, Peter H. King

**Affiliations:** 1 Department of Neurology, University of Alabama at Birmingham, Civitan 545C, 1530 3rd Avenue South, Birmingham, AL 35294 USA; 2 Department of Cell, Developmental, and Integrative Biology, University of Alabama at Birmingham, Birmingham, AL 35294 USA; 3 Department of Medicine (Division of Nephrology and Hypertension), University of Alabama at Birmingham, Birmingham, AL 35294 USA; 4 Birmingham Veterans Affairs Medical Center, Birmingham, AL 35294 USA; 5 Department of Neurology, University of Miami, Miami, FL 33136 USA

**Keywords:** Amyotrophic lateral sclerosis, Biomarkers

## Abstract

Amyotrophic lateral sclerosis (ALS) is a fatal neurodegenerative disease characterized by progressive muscle weakness. Skeletal muscle is a prime source for biomarker discovery since it is one of the earliest sites to manifest disease pathology. From a prior RNA sequencing project, we identified FGF23 as a potential muscle biomarker in ALS. Here, we validate this finding with a large collection of ALS muscle samples and found a 13-fold increase over normal controls. FGF23 was also increased in the SOD1^G93A^ mouse, beginning at a very early stage and well before the onset of clinical symptoms. FGF23 levels progressively increased through end-stage in the mouse. Immunohistochemistry of ALS muscle showed prominent FGF23 immunoreactivity in the endomysial connective tissue and along the muscle membrane and was significantly higher around grouped atrophic fibers compared to non-atrophic fibers. ELISA of plasma samples from the SOD1^G93A^ mouse showed an increase in FGF23 at end-stage whereas no increase was detected in a large cohort of ALS patients. In conclusion, FGF23 is a novel muscle biomarker in ALS and joins a molecular signature that emerges in very early preclinical stages. The early appearance of FGF23 and its progressive increase with disease progression offers a new direction for exploring the molecular basis and response to the underlying pathology of ALS.

## Introduction

Amyotrophic lateral sclerosis (ALS) is a neurodegenerative disorder characterized by progressive motor neuron loss and skeletal muscle weakness. Biomarkers hold great promise to differentiate ALS from clinical mimics (diagnostic), select the subset of patients most likely to benefit from a particular treatment (predictive), predict future course of disease (prognostic), quantify treatment response (pharmacodynamic), or inform disease biology (biotype)^1,2^. Significant progress has been made in showing that markers of axonal degeneration have prognostic value as well as potential utility in demonstrating pharmacodynamic effect^3–5^. There remains, however, a real need for biotype markers that inform the underlying biology of disease, especially if these have potential to differentiate patients in whom heterogeneous biological mechanisms might be at play. In turn, such insights could inform patient selection for clinical trials that target the relevant biology. Skeletal muscle, the end-organ responsible for the progressive weakness that defines ALS, is an accessible tissue with great potential to facilitate discovery of novel biomarkers. Moreover, prior studies indicate that the earliest pathological changes in ALS occur peripherally at the level of skeletal muscle and the neuromuscular junction^6–8^. Early involvement of skeletal muscle is underscored by our prior work which defines a molecular signature in ALS skeletal muscle beginning in the earliest pre-clinical phases of disease based on correlative studies with the SOD1^G93A^ mouse. Characterization of this signature emerged from RNA sequencing of human ALS muscle, and encompasses genes of diverse pathways including Smads, TGF-β, vitamin D (CYP27-B1), FRZB/Wnt signaling, and select microRNAs^9–13^. Some of the markers, such as Smad 1, 5, 8, and TGF-β, appear to be specific for ALS whereas FRZB and CYP27B1 were increased in non-ALS neurogenic processes. In this report, we characterize FGF23, a gene identified in the original human RNA sequencing project, as part of this molecular signature. Our findings broaden the scope of potential signaling pathways in muscle that are activated early in the course of ALS through end-stage, and provide direction for the development of new treatments.

## Results

### FGF23 mRNA is upregulated in ALS muscle tissue

**Figure 1:**
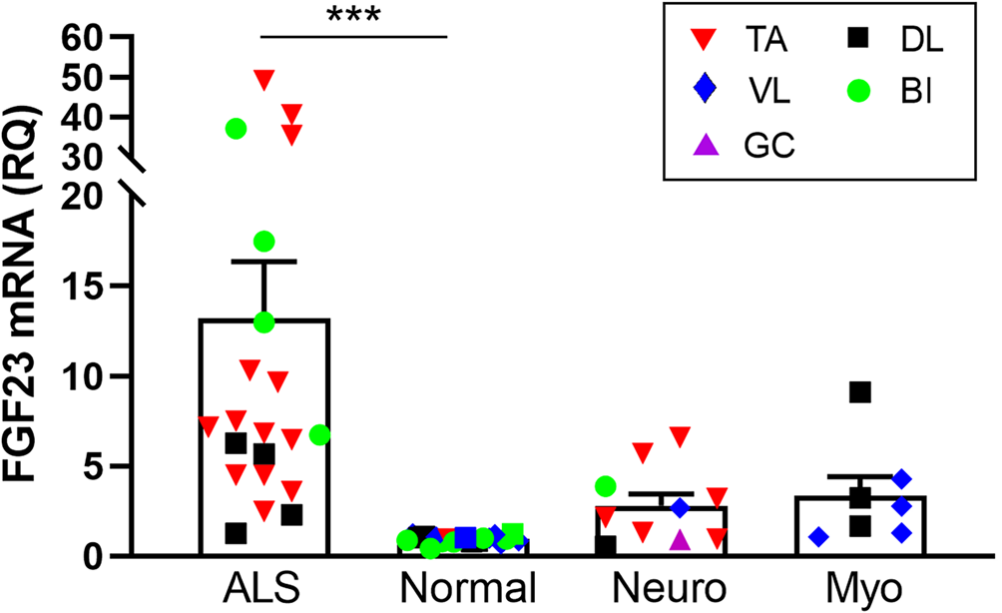
*FGF23* mRNA levels are increased in ALS muscle tissue. *FGF23* mRNA levels were assessed in human muscle samples by qPCR using GAPDH as an internal housekeeping control. Disease samples were expressed as a fold-change (mean ± SEM) compared to normal control tissue (set at 1). ***P = 0.0001. BI, biceps brachii; DL, deltoid; GC, gastrocnemius; Myo, myopathy disease control; neuro, neuropathy disease control, TA, tibialis anterior; VL, vastus lateralis.

*FGF23* was identified in an earlier RNA sequencing project comparing transcriptomes between normal and ALS muscle samples using small sample numbers^11^. To validate this finding, we expanded our sampling and included muscle biopsy samples from neuropathy and myopathy disease controls (Table 1). The demographics of our ALS population were consistent with those we and others previously published^14,15^. Age and gender of normal subjects were reasonably matched whereas disease control groups were matched to age but had differences in gender ratio (higher number of males in the neuropathy group versus a higher number of females in the myopathy group). With these samples, we first assessed *FGF23* mRNA levels by qPCR and found a 13 -fold increase in ALS muscle samples over normal control subjects (*P* = 0.0001; Fig. 1). The values were variable, going as high as ~ 50-fold over the mean for the normal control group. Values for the neuropathy and myopathy disease control groups trended slightly higher than normal controls but did not reach significance. It should be noted that muscle selection for control diseases was generally based on their clinical patterns of weakness (i.e. proximal muscles for myopathy and distal muscles for neuropathy) and this may have affected the comparisons. In summary, these findings show a marked and significant increase in *FGF23* mRNA levels in ALS muscle samples.

**Table 1:** Demographic and clinical data of muscle biopsy cohorts.

	Normal	ALS	Myopathy	Neuropathy
Number	20	21	7	10
Mean age (years)^a^	53 ± 14	61 ± 13	54 ± 14	61 ± 17
Age range (years)	15–77	33–86	35–82	33–88
Gender (M:F)	11:9	9:12	1:6	8:2
Duration^b^ (months)		10 ± 6		
Clinical phenotype		Spinal onset (14 )	Inflammatory (4)	Non-specific (5)
		Bulbar onset (6)	Non-specific (1)	CIDP (1)
		Unknown (1)	Mitochondrial (1)	Plexopathy (3)
			Metabolic (1)	Axonal GBS (1)
**Muscle biopsy**				
Biceps brachii	8	4	–	1
Deltoid	3	4	3	1
Vastus lateralis	8	–	4	1
Tibialis anterior	1	13	–	6
Gastrocnemius				1

*CIDP* chronic inflammatory demyelinating polyradiculoneuropathy, *GBS* Guillain Barre syndrome, *SD* standard deviation.

^a^Mean age (± SD) at time of sample collection.

^b^Mean duration (± SD) from onset of symptoms to sample collection. Duration was unknown in three patients.

### FGF23 is detected in the endomysial connective tissue of ALS muscle samples

**Figure 2:**
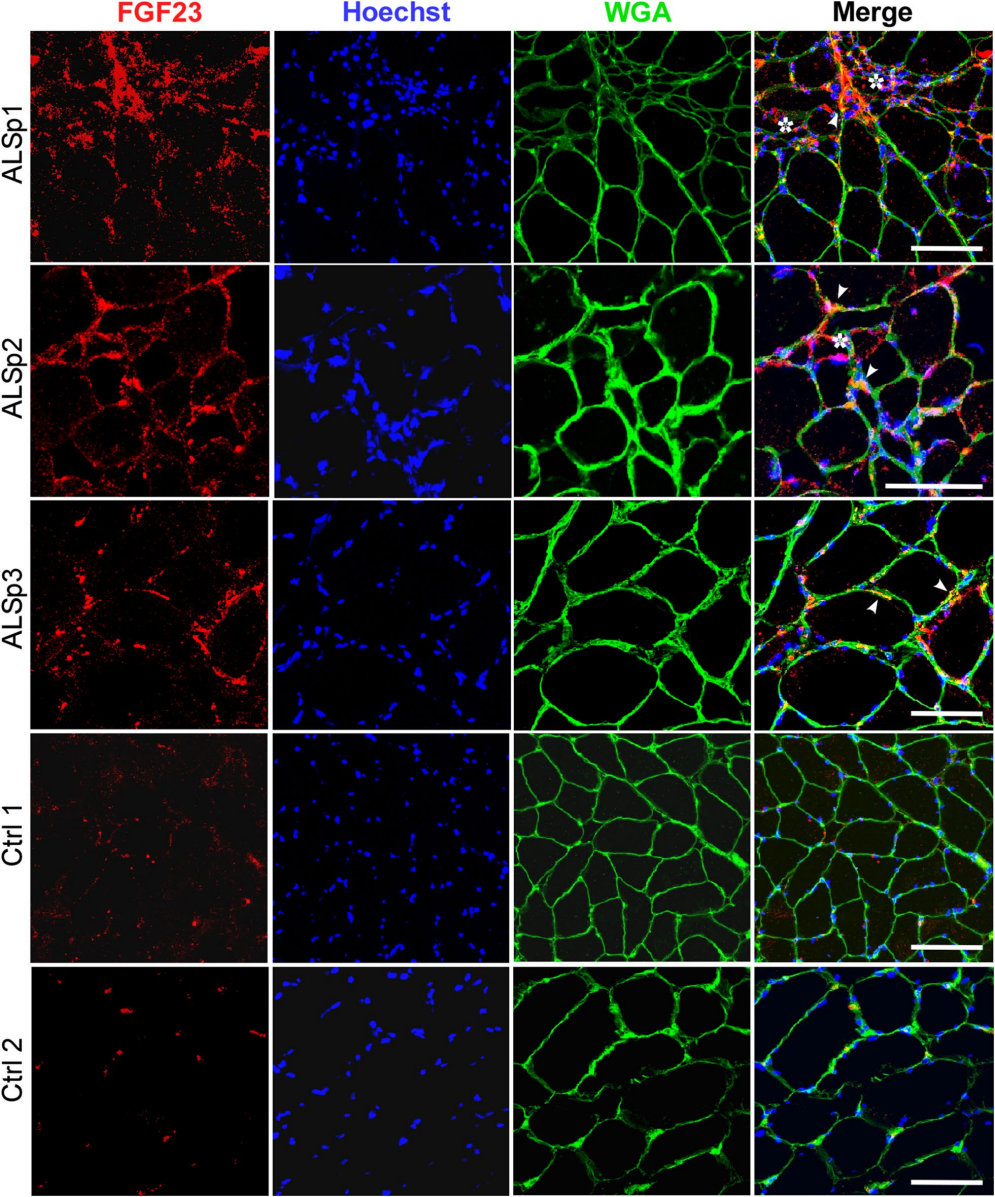
FGF23 protein is increased in ALS muscle tissue. Sections from 6 ALS and 4 normal muscle biopsy samples were immunostained with an anti-FGF23 antibody and counterstained with Hoechst and wheat germ agglutin (WGA). All 6 ALS patient samples but no control samples showed positive staining. Three of the ALS and two of the normal control sections are shown here. ALSp1 (deltoid), ALSp2 (vastus lateralis), ALSp3 (vastus lateralis), Ctrl1 (deltoid), Ctrl 2 (vastus lateralis). Asterisks highlight areas of grouped atrophy and arrowheads highlight several of the loci where FGF23 and WGA immunostaining colocalizes. Scale bars, 100 μm.

**Figure 3:**
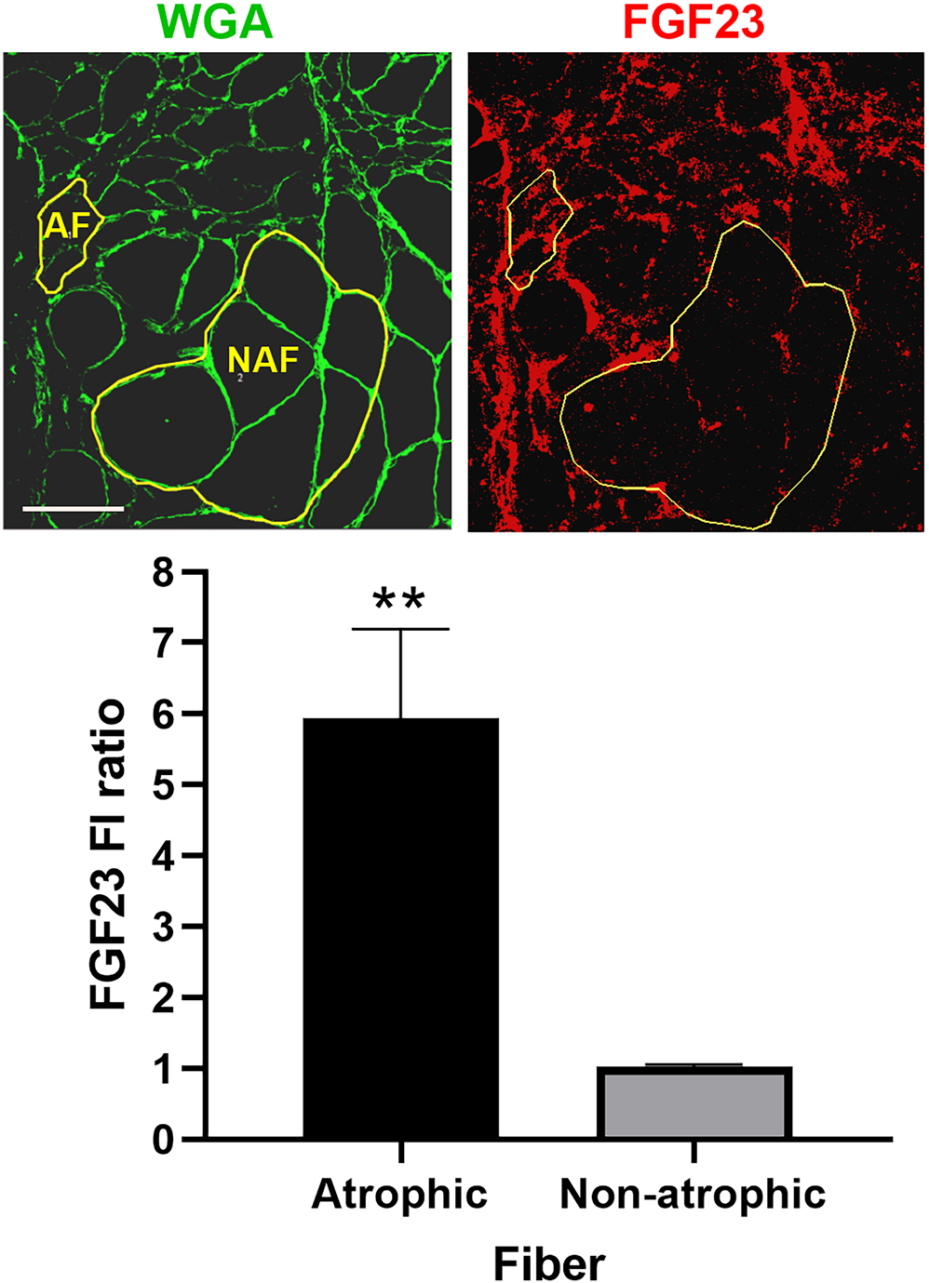
FGF23 immunoreactivity is higher in areas of grouped atrophy in human ALS muscle tissue. Using ImageJ Fluorescence Intensity (FI) Analysis function, we compared the FGF23 FI (per μM^2^) of 37 atrophic fibers (< 25 μM minimal feret’s diameter) sampled from areas of grouped atrophy seen in 5 human ALS muscle samples with 37 non-atrophic fibers (> 25 μM minimal feret’s diameter) from the same sections. Representative photomicrographs of patient ALSp1 are shown with regions of interest highlighted for 6 grouped atrophic fibers (AF) and 6 non-atrophic fibers (NAF). An FGF23 intensity ratio was calculated by dividing the FI in atrophic fibers by the FI in non-atrophic factors for each patient. A ratio was also calculated between a similar number of non-atrophic fibers in the same section to the NAF region of interest as a control. The FI ratio was nearly sixfold higher in areas of grouped atrophy versus non-atrophic fibers. **P = 0.006. Scale bar, 100 μm.

Having validated the consistent upregulation of FGF23 mRNA in muscle lysates of ALS muscle samples, we performed immunohistochemistry to determine the pattern of expression in human muscle tissue. We assessed FGF23 immunoreactivity in muscle biopsy sections from 6 ALS patients and 4 normal subjects. We detected FGF23 immunoreactivity in all 6 ALS patients, three of which are shown in Fig. 2. There was diffuse and extensive immunoreactivity in the endomysial connective tissue surrounding the myofibers with some punctate foci on the muscle membrane as indicated by colocalization with wheat germ agglutinin (WGA). With secondary antibody alone, there was no signal detected with ALSp1 which showed the strongest FGF23 immunoreactivity (Supplemental Fig. S1). Areas of grouped atrophy, as characterized by aggregations of small and angular fibers (ALSp1 and ALSp2 muscle sections), appeared to have higher FGF23 fluorescence intensity (FI). To determine whether there was an association between FGF23 FI and atrophied fibers, we assessed regions of interest (ROI) for FGF23 FI (per μM^2^) in equal numbers of non-atrophic and atrophic fibers within the same muscle section (Fig. 3). We calculated an FGF23 FI ratio (atrophic to non-atrophic fibers), as this approach controlled for variable staining intensity between different patient samples. In 5 ALS patients, we found that the mean FI ratio was nearly sixfold higher in areas of grouped atrophy versus matched numbers of non-atrophic fiber groups (*P* = 0.006). Only minimal punctate immunoreactivity was detected in muscle sections of normal control subjects (Ctrl 1 and Ctrl 2 are shown as examples). Overall, FGF23 immunoreactivity was observed consistently in ALS denervated muscle, localized mainly to the endomysial connective tissue and muscle membrane, with significantly higher intensity in areas of grouped atrophy.

### FGF23 is elevated in the SOD1^G93A^ mouse and tracks disease progression

**Figure 4:**
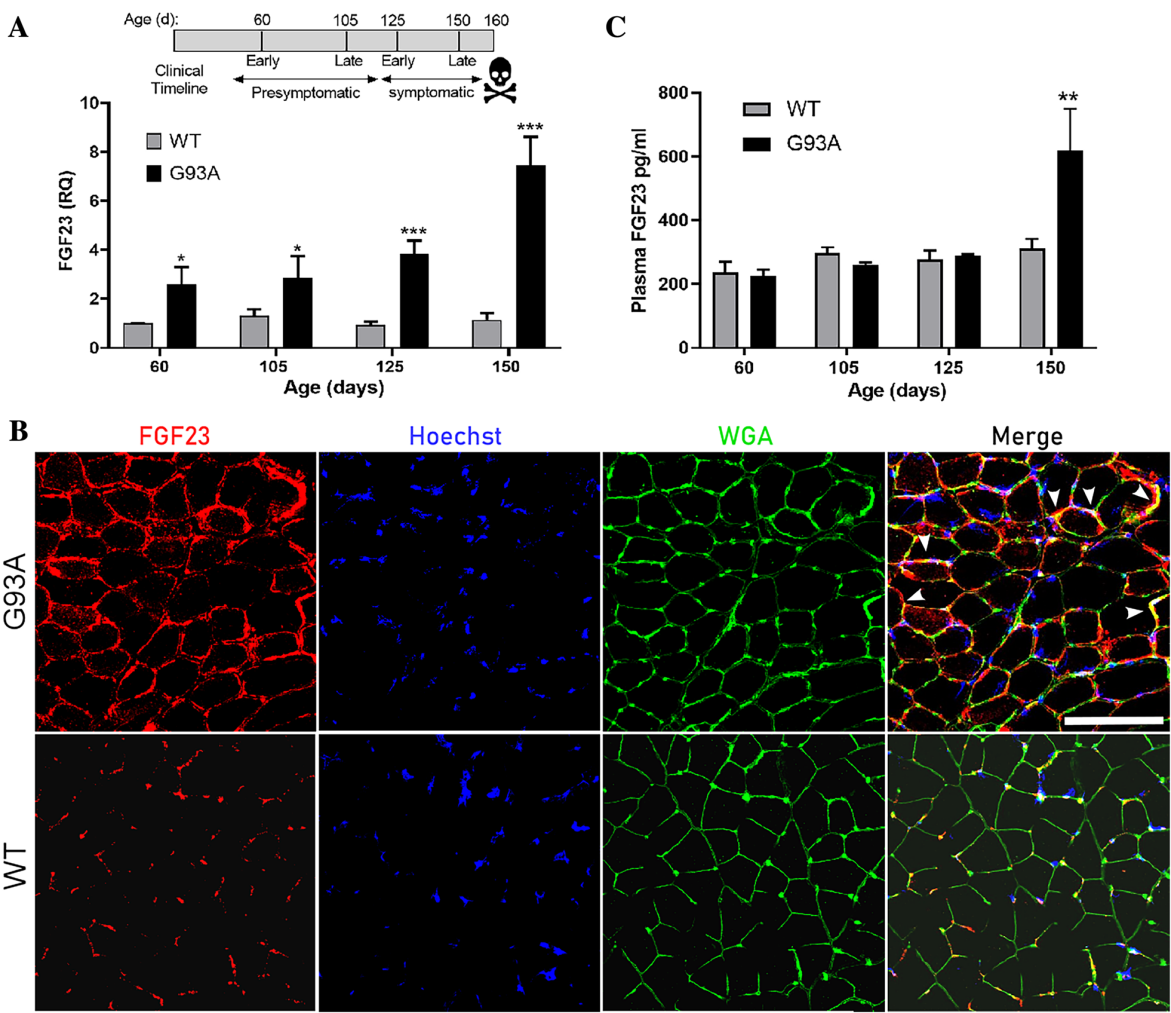
FGF23 is increased in SOD1^G93A^ muscle. (**A**) The clinical timeline of disease progression in the SOD1^G93A^ mouse is shown above^11^. Below is a qPCR analysis of gastrocnemius muscle samples from littermate controls (WT) and SOD1^G93A^ mice at different ages as indicated. Data points are the mean ± SEM of 6–8 mice. *P < 0.05, ***P < 0.0005. (**B**) Photomicrographs of gastrocnemius muscle sections from a WT and SOD1^G93A^ mouse (60 d) immunostained with an anti-FGF23 antibody and counterstained with Hoechst and WGA. Scale bar, 100 μm. Arrowheads highlight several areas of merged FGF23 and WGA staining. (**C**) ELISA analysis of FGF23 in plasma samples obtained at the ages indicated. Data points are the mean ± SEM of 3 mice per group. **P < 0.01.

Although patients with *SOD1* mutations only represent ~ 2% of the ALS population, the SOD1 mouse model reflects many of the clinicopathological changes that occur in ALS^16^. It provides an opportunity to examine temporal patterns in tissue biomarker expression pre-clinically, at disease onset, and throughout the disease course. Here, we assessed the temporal pattern of *FGF23* mRNA expression in the *SOD1*^G93A^ mouse. In this model, we have previously used declines in rotarod performance and weight measurements to determine onset of symptoms^11^. Here, we sampled the gastrocnemius muscle at different time points (days 60, 105, 125, and 150) reflective of this clinical timeline (Fig. 4A). In SOD1^G93A^ mice, *FGF23* mRNA was significantly elevated at a very early and presymptomatic age (60 d) compared to littermate controls (WT). This difference progressively increased to nearly eightfold by end-stage (150 d). This temporal pattern is similar to what we previously observed with other muscle biomarkers in this model^10–13^. To determine the localization of FGF23 expression in SOD1^G93A^ muscle tissue, we immunostained gastrocnemius muscle sections with an anti-FGF23 antibody and found a pattern of immunoreactivity similar to human ALS muscle tissue (Fig. 4B). There was diffuse immunoreactivity in the endomysial connective tissue and muscle membrane from muscle sections in the SOD1^G93A^ mouse but only scant, punctate reactivity in WT muscle. We next determined if FGF23 could be detected by ELISA in plasma samples of SOD1^G93A^ mice (Fig. 4C). Only at end-stage (day 150) was there a significant ~ twofold increase in plasma FGF23 levels in the SOD1^G93A^ mice (*P* < 0.01). Taken together, FGF23 is increased in the early pre-clinical stage of disease in a pattern similar to human ALS tissue and markedly increases toward end-stage. Only at end-stage, however, can FGF23 be detected in the peripheral circulation.

### FGF23 is not increased in human ALS plasma samples

**Figure 5:**
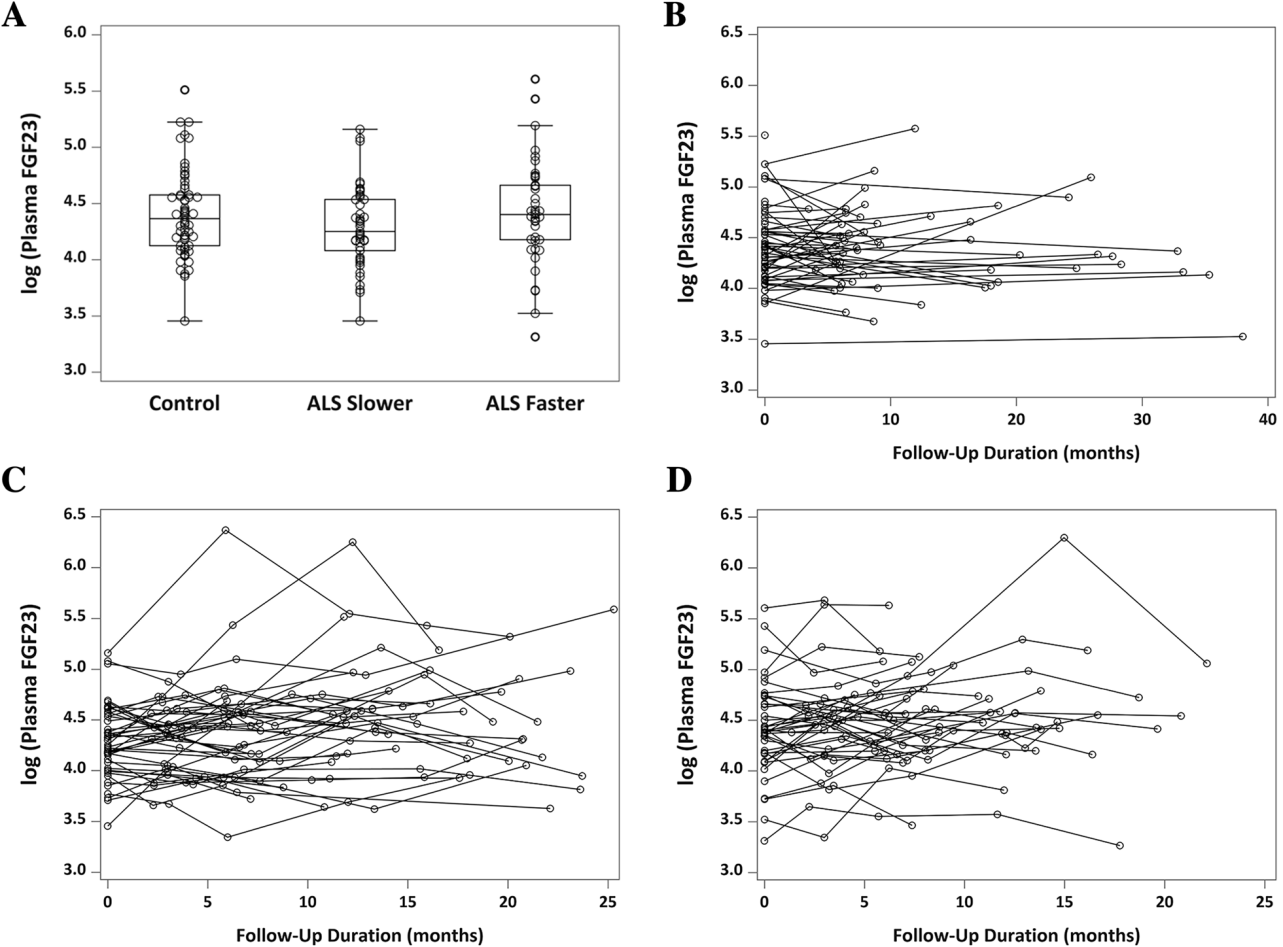
FGF23 in human plasma samples from ALS patients and healthy controls. (**A**) Baseline levels of log-transformed plasma FGF23 concentration (pg/ml) among controls, and faster and slower progressing ALS patients. Boxes show median (middle line), and 25th and 75th percentiles (lower and upper border, respectively); whiskers extend to a maximum of 1.5 × interquartile range (IQR), or to the most extreme value if it is less than 1.5 × IQR from the 25th or 75th percentile. (**B**) Longitudinal changes in log-transformed plasma FGF23 among controls. (**C**) Longitudinal changes in log-transformed plasma FGF23 among ALS slower progressors (ALSFRS-R decline < 0.8 point/month). (**D**) Longitudinal changes in log-transformed plasma FGF23 among ALS faster progressors (ALSFRS-R decline > 1.2 points/month).

Plasma FGF23 levels were evaluated in 86 ALS patients who had been pre-selected as slower (n = 45) or faster (n = 41) progressors based on a prospectively measured ALSFRS-R decline of less than 0.8 point/month or greater than 1.2 points/month, respectively, as well as in 61 healthy age- and sex-matched controls. Demographic and clinical characteristics of the study population are summarized in Table 2. Plasma FGF23 concentrations at baseline did not differ between controls, slower progressors and faster progressors (*P* = 0.19; Table 3; Fig. 5A). With follow-up durations spanning a median (range) of 8 (3.5–38) months among controls, 16.1 (6–25.3) months among slower progressors, and 10.7 (4.5–22.1) months among faster progressors, plasma FGF23 levels were largely stable over time in each group (Fig. 5B–D).

**Table 2:** Study participant characteristics.

		Control (N = 61)	ALS Slower Progressor (N = 45)	ALS Faster Progressor (N = 41)
# of plasma collections	1 2 3 4 5	6 55 – – –	– – 13 14 18	– – 22 9 10
Total follow-up duration (months)	Median (range)	8.0 (3.5–38.0)	16.1 (6.0–25.3)	10.7 (4.5–22.1)
Baseline age (years)	Mean ± SD (range)	53.7 ± 11.3 (28–84)	56.0 ± 11.2 (30–86)	59.9 ± 7.4 (47–81)
Male	N (%)	26 (43%)	21 (47%)	20 (49%)
Genotype	*SOD1* *C9orf72* Unknown	(n/a)	3^a^ 10 32	0 5 36
Clinical diagnosis	ALS ALS-FTD		43 2	39 2
Site of onset	Bulbar Limbs Other^b^ Mixed		5 35 1 4	8 24 0 9
Years from onset to baseline	Median (range)		3.3 ± 3.6 (0.6–19.7)	1.4 ± 0.8 (0.5–3.7)
Years from diagnosis to baseline	Median (range)		1.6 ± 2.1 (0.0–9.7)	0.7 ± 0.6 (0.1–2.7)
Baseline ALSFRS-R	Mean ± SD (range)		35.1 ± 7.6 (11–47)	37.3 ± 5.8 (23–46)
Baseline deltaFRS^c^ (point(s) per month)	Mean ± SD (range)		− 0.5 ± 0.4 (− 2.3, 0.0)	− 0.7 ± 0.5 (− 2.2, − 0.2)
ALSFRS-R slope^d^ (point(s) per month)	Mean ± SD (range)		− 0.3 ± 0.3 (− 0.8, 0.2)	− 1.8 ± 0.5 (− 3.1, − 1.2)

Baseline = first visit at which plasma sample was available. Total follow-up duration = time between the participant’s first and last plasma sample included in this study.

(n/a) = not applicable.

^a^The 3 SOD1 variants were A89V, E100K, and E121G.

^b^Site of onset other than bulbar or limbs (e.g. frontotemporal, respiratory).

^c^ALSFRS-R rate of decline from time of onset (assuming ALSFRS-R = 48) to baseline; negative value indicates decline.

^d^Negative value indicates decline.

**Table 3:** Plasma FGF23 concentration.

		Control^b^	ALS slower progressor	ALS faster progressor
		(N = 60)	(N = 45)	(N = 41)
**Baseline only**				
Original scale (pg/ml)	Median (range)	78.7 (31.7–247.1)	70.2 (31.7–174.2)	81.6 (27.5–271.8)
Log-transformed^a^	Mean ± SD (range)	4.40 ± 0.39 (3.46–5.51)	4.29 ± 0.36 (3.46–5.16)	4.42 ± 0.46 (3.31–5.61)

		(114 visits)	(185 visits)	(152 visits)
**Baseline and follow-ups**				
Original scale (pg/ml)	Median (range)	78.1 (31.7–263.6)	79.6 (28.38–583.2)	84.7 (26.22–543.17)
Log-transformed^a^	Mean ± SD (range)	4.40 ± 0.38 (3.46–5.57)	4.39 ± 0.46 (3.35–6.37)	4.47 ± 0.47 (3.27–6.30)

Baseline = first visit at which plasma sample was available.

N = number of participants. Visits = number of person-visits.

^a^Natural algorithm.

^b^N = 1 control whose FGF23 values were extreme outliers (773.5 and 988.3 pg/ml at baseline and follow-up, respectively) was excluded from table above.

## Discussion

The identification of FGF23 as a potential muscle marker of ALS emerged from our RNA sequencing comparison of ALS and normal muscle biopsies^11^. This finding was unexpected based on the well-established role of FGF23 in regulating systemic calcium and phosphate metabolism^17^. In this report we have shown that FGF23 expression is increased in ALS muscle tissue and progressively increases with disease progression in the SOD1^G93A^ mouse model of ALS. It is localized mainly to the endomysial connective tissue and muscle membrane, but increases are not detected in plasma samples off ALS patients. This report identifies skeletal muscle as a potential extra-renal target of FGF23 and raises important questions as to its role in ALS disease pathology.

While FGF23 has been detected in other organs such as brain, heart and thymus, only very low levels are present in murine and human skeletal muscle^18,19^. The basis (and source) for increased FGF23 expression in skeletal muscle in our study is unclear but may relate to the inflammatory response present in the peripheral motor system of ALS patients. During disease progression, activated macrophages infiltrate the peripheral motor system, including skeletal muscle, and they have been shown in other tissues to produce FGF23 in response to inflammatory cytokines such as TNF-α and IL-1β^20–22^. We have shown previously that the inflammatory milieu in ALS muscle is further enriched by the accumulation of activated mast cells and neutrophils that also secrete FGF23-inducing cytokines^23–26^. Since FGF23 itself is pro-inflammatory and can induce macrophages to produce inflammatory cytokines, a positive feedback loop may be at play^22^. Lastly, TGF-β2 can directly induce FGF23 expression, and we identified elevated levels of this cytokine in human ALS muscle in the same time frame as FGF23 in the SOD1^G93A^ mouse^13^ . Although a prior report suggests that myofibers express FGF23, we were not able to detect intra-fiber staining^27^. this may relate to a higher specificity of the antibody used in our studies. Interestingly, in cardiac tissue, FGF23 promotes activation of fibroblasts and fibrosis in the presence of TGF-β1, a cytokine that is elevated in ALS muscle and increases with disease progression^13,28^. Fibrosis is substantial in ALS muscle tissue with evidence linking it to TGF-β1 signaling^29,30^.

While our report clearly demonstrates increased FGF23 in the endomysial connective tissue and muscle membrane, a key question is whether FGF23 signaling is active in ALS muscle fibers. Myoblasts and myotubes have been shown to express FGF23 receptors (FGFr1-4) and Klotho, the coreceptor/cofactor necessary for FGF23 receptor binding and activation, although direct activation of this signaling pathway has not yet been demonstrated in normal muscle tissue^31,32^. One well-described effect of FGF23 signaling is suppressed transcription of CYP27B1, the major enzyme that activates vitamin D, in renal and extrarenal tissues^33,34^. This downstream effect would be at odds with our prior report showing significant upregulation of myofiber CYP27B1 in ALS disease progression^12^. Further mechanistic studies understanding the roles of FGF23 and vitamin D at the level of skeletal muscle will be required to help reconcile this juxtaposition.

Although the role of FGF23 in promoting inflammation in diseases such as chronic kidney or lung disease^35–37^, might suggest a deleterious role in ALS patients, one report observed that ectopic FGF23 can improve aspects of mitochondrial function in skeletal muscle^31^. Its upregulation in ALS may therefore represent an attempt to compensate for mitochondrial dysfunction in skeletal muscle that starts at the very earliest stages of disease pathology^38^. On the other hand, FGF23 can reduce the population of mesenchymal stem cells in muscle, induce their senescence, and impair the regenerative/reparative capacity of the muscle^39^.

The rationale for pursuing FGF23 as a biomarker in ALS was based on its progressive increase in skeletal muscle with disease progression in the SOD1^G93A^ mouse and its properties as a secreted factor. Unfortunately, we were unable to detect any changes in the plasma of ALS patients (and only at the end stage in the SOD1^G93A^ mouse) thus limiting its utility in the clinic. Our investigation was thorough and included a large number of ALS patients representing the diversity of clinical phenotypes and a large age-matched control group. Several possibilities may explain this negative result. First, the amount of FGF23 produced in skeletal muscle, even with disease progression, may be too small to detect peripherally if some entered the circulation. Second, FGF23 also has a relatively short half-life in circulation and so increases may not be detected if release of FGF23 is not constant^40^. Third, and more intriguingly, FGF23 may be trapped in the endomysial compartment based on its structural properties. Along with other members of the FGF family, FGF23 has a heparan sulphate glycosaminoglycan (HSGAG) binding site which can bind to HSGAGs through electrostatic interactions^33^. HSGAGs are enriched in the basal lamina surrounding muscle fibers and may serve to facilitate FGF signaling by anchoring the ligand at or near the cell surface^33,41,42^. Our immunostaining patterns showing FGF23 at or near the muscle membrane are supportive of this possibility (Figs. 2 and 4). Heparan sulfate binding is required for proper formation of the Klotho/FGF23/FGFr complex and FGFr activation^43^, and loss of HSGAGs at the cell surface (e.g. in kidney cells) can abrogate FGF23 activity^44^. Interestingly, we observed a similar pattern with FRZB, a secreted Wnt antagonist, which is also associated with grouped atrophic fibers, increases in the endomysial compartment with ALS disease progression, and cannot be detected in the peripheral circulation^9^. Although FRZB does not have a defined HSGAG binding site, it has an overall net positive charge at physiological pH similar to FGF23 (+ 7.454 versus + 5.971), and thus may also associate with negatively charged HSGAGs. The explanation for why we could detect FGF23 by ELISA in the SOD1^G93A^ mouse and not humans is not immediately clear but may have been related to the end-stage of disease where mice are moribund. Potential FGF23 triggers such as inflammatory cell infiltration or TGF-β2 are at maximum levels in skeletal muscle at this stage^13,24^. It may also relate to dehydration or other metabolic disturbances at end-stage which may be a trigger for systemic FGF23 production^45^.

In summary, this report validates FGF23 as a new skeletal muscle biomarker in ALS. It broadens the molecular signature of muscle biomarkers that appear in early pre-clinical stages and increase with disease progression, reflecting a diverse and complex physiological or pathophysiological response to ALS. This report also illustrates that the search for biomarkers can open up new directions for understanding disease pathology and therapeutic development without being immediately translatable to the clinic. Interestingly, FGF23 has been identified as a biomarker of aging, frailty, and age-related diseases including neurodegeneration (dementia) and sarcopenia^46–49^. Our findings identify ALS as another neurodegenerative disease linked to FGF23, and it will be of great interest in future studies to determine its contribution to disease progression.

## Methods

### Human tissue and plasma

Human ALS and control muscle samples were selected from the archive of remnant muscle biopsy tissues at the UAB Division of Neuromuscular Disease as previously detailed^9^. All patients were older than 18 years and were consented according to the protocol approved by the UAB Institutional Review Board (IRB). Autopsy muscle samples were obtained from UAB patients who were enrolled in an ALS tissue collection program directed by PHK. Patients were older than 18 years and were consented based on a research protocol approved by the UAB IRB. All ALS patients who underwent muscle biopsy were eventually diagnosed with definite ALS as defined by the revised El Escorial criteria. Most of these patients were followed in the clinics at UAB. Normal control muscle biopsy samples were from patients with non-specific muscle symptoms such as pain but were interpreted as normal by a neuromuscular pathologist. Neuropathy and myopathy samples were chosen based on histological evidence of denervation or myopathy, as determined by a pathologist, in conjunction with clinical history and electrophysiological testing.

Human blood samples: ALS patient samples were derived from the CReATe Consortium’s *Phenotype-Genotype-Biomarker (PGB)* study (NCT02327845). For this experiment, only study participants with plasma samples available at three or more study visits; no renal failure; and a prospectively measured ALSFRS-R rate of decline of < 0.8 point/month (slower progressor group) or > 1.2 points/month (faster progressor group) were considered for inclusion. Control samples were collected at the University of Miami through the CRiALS Study (NCT00136500) and at the University of Alabama at Birmingham. The 3 groups were matched on age and sex. All participants were older than 18 years and provided informed consent under research protocols approved by the University of Miami and the University of Alabama at Birmingham IRBs. Blood samples were drawn, and centrifuged at × 1600 RCF for 10 min at 4 °C. The liquid component (plasma) was immediately transferred to clean vials, and stored at − 80 °C for further analysis. Samples sizes chosen were based on prior testing of other targets identified by the original RNA sequencing project where the fold-difference between control and ALS samples was similar^12^. All experimental methods human subjects and samples were carried out in accordance with relevant guidelines and regulations.

### Animals

All animal procedures were approved by the UAB Institutional Animal Care and Use Committee and were carried out in accordance with relevant guidelines and regulations of the National Research Council Guide for the Care and Use of Laboratory Animals and in compliance with the ARRIVE guidelines. B6.Cg-Tg (SOD1_G93A) 1 Gur/J male mice (The Jackson Laboratory) were bred with C57BL/6J females to generate hemizygous SOD1^G93A^ offspring with wild-type (WT) littermates as previously detailed. These mice have later clinical onset and prolonged survival compared to the original ALS model on the B6/SJL background^50^. After sacrifice by CO_2_ inhalation followed by cervical dislocation, gastrocnemius muscle tissue samples were collected from SOD1^G93A^ and WT littermate controls at post-natal day 60, 105, 125 and 150 as previously described^9,12^. These time points cover the full range of disease stages in the ALS mouse: early pre-symptomatic (60 d), late pre-symptomatic (105 d), early symptomatic (125 d), and late symptomatic (150 d) based on rotarod and weight testing as previously described^11^.

Mouse blood samples were collected by retro-orbital puncture. Approximately 200ul blood per mouse were put into a Sigmacote (Sigma) treated tube containing 20ul of anticoagulant, placed on ice for up to 30 min, and then centrifuged at 4 °C for 30 min at 1000 g. Supernatants were transfered to fresh siliconized tubes and stored at − 80° C. Samples sizes were chosen based on prior testing of other targets identified by the original RNA sequencing project where the fold-difference between control and ALS samples was similar^11^.

### RNA analysis

Two micrograms of RNA were reverse-transcribed using the High-Capacity cDNA Reverse Transcription Kit (Applied Biosystems) and FGF23 mRNA expression was quantified by Taqman real-time PCR (Applied Biosystems). GAPDH expression was used as an internal control as described previously.

### Immunohistochemistry

For immunohistochemistry, muscle samples were embedded in a mixture of tragacanth gum/OCT and flash frozen in an isopentane bath over liquid nitrogen. Muscle tissues were cut into 10 μm sections and air-dried at room temperature for 20 min followed by fixation with cold acetone for 3 min at − 20 °C. Slides were incubated in 3% hydrogen peroxide for 10 min. After blocking, slides were incubated with FGF23 antibody for human (21-6610, 1:100, Quidel) and antibody for mouse (21-6320, 1:500, Quidel) overnight at 4 °C. After washing in PBS, slides were incubated with HRP secondary antibody (Vector Laboratories) for 90 min at RT, followed by TSA Cy3 (PerkinElmer, Waltham, MA) for 30 min. Sections were incubated in Wheat Germ Agglutinin (WGA), Oregon Green 488 Conjugate (ThermoFisher), followed by Hoechst 33342 (Sigma- Aldrich, St. Louis, MO) at 1:20,000 for 5 min. Slides were imaged using a Nikon C2 confocal microscope.

### ELISA

Human plasma FGF-23 was analyzed using U-PLEX Human FGF-23 Assay (K1516EK, MSD), and mouse plasma FGF23 was measure using mouse/rat FGF23 (intact) ELISA kit (60-6800, Quidel) according to the manufacturer’s instruction.

### FGF23 fluorescence intensity analysis

For assessment of immunoreactive FGF23 fluorescent intensity (FI) versus myofiber size, we used the FI Analysis function in ImageJ 1.53c (National Institute of Health). Myofiber size was determined using the minimal Feret’s diameter and fibers with diameters of less than 25 μm were considered atrophic. FI was quantified as unit of FGF23 FI per μm^2^. We compared a total 37 atrophic fibers to a matched number of non-atrophic fibers from the same sections from five ALS patients. The atrophic fibers were all sampled from areas of grouped atrophy. We observed minimal variations in the FI ratio in non-atrophic fibers, and the ratio of normal to normal within the same section came to the expected value of ~ 1 (range 0.92–1.12).

### Statistics

Statistical analyses for human tissue and mouse data were performed in Graphpad Prism 8. A one-way ANOVA with Dunnett’s multiple comparison was used to assess FGF23 mRNA expression in human muscle biopsy tissues. A student’s t test was performed for mouse qPCR and ELISA data, comparing littermate control to SOD1^G93A^ mice for each age. An unpaired t test was used comparing FGF23 FI between atrophic and non-atrophic fibers. For human plasma data, statistical analysis was performed, and summary statistics and figures generated, using SAS 9.4. Natural logarithm transformations were applied to FGF23 values to reduce data skewness. One control participant whose FGF23 concentrations were extreme outliers was excluded from analysis and figures. Baseline FGF23 levels were compared between groups by one-way ANOVA. The level of statistical significance was set at 0.05 (two-sided).

## Supplementary Information

Supplementary Figure S1.
