# Supplementary material for: FGF23, a novel muscle biomarker detected in the early stages of ALS
Source: Sci Rep. 2021 Jun 8;11:12062. doi: 10.1038/s41598-021-91496-6 (PMC8187665; doi:10.1038/s41598-021-91496-6)
Supplement: Supplementary file 1 — Supplementary Figure S1. [file 41598_2021_91496_MOESM1_ESM.pdf]

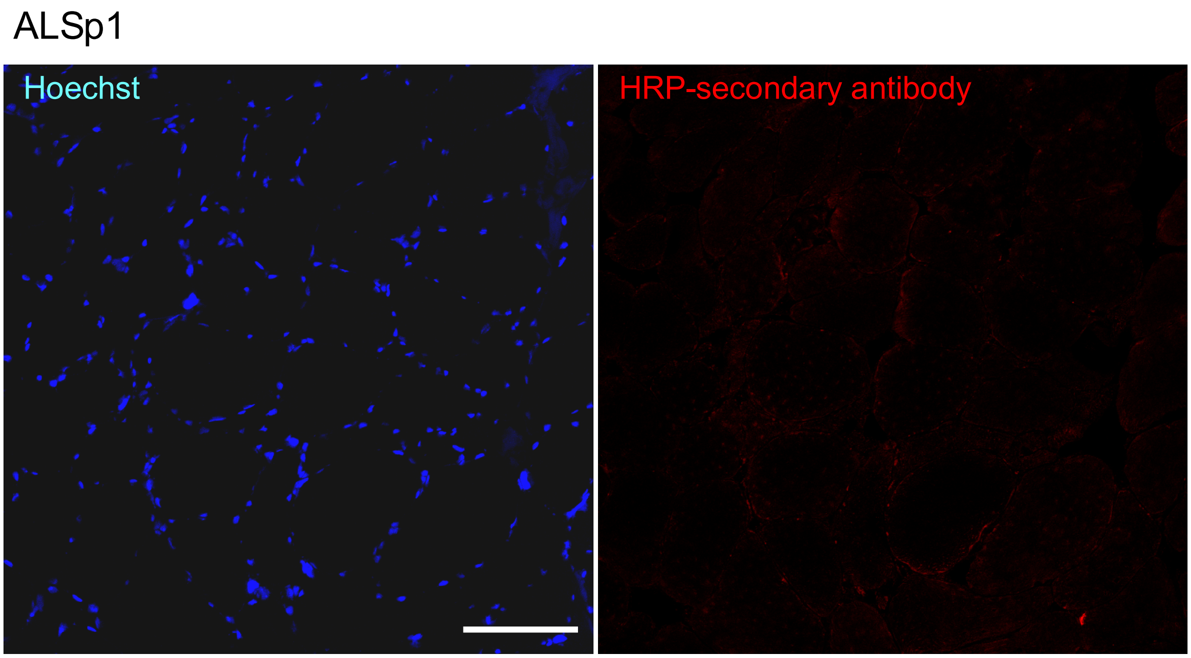

Supplemental Fig. S1. Negative control for FGF23 immunostaining for ALSp1 shown in Fig. 2. A muscle tissue section from patient ALSp1 was probed with HRP-conjugated secondary antibody without the FGF-23 primary antibody, followed by TSA Cy3 for 30 min. Scale bar, 100 μM.
